# Supplementary material for: Green Synthesis of Characterized Silver Nanoparticle Using Cullen tomentosum and Assessment of Its Antibacterial Activity
Source: Antibiotics (Basel). 2023 Jan 18;12(2):203. doi: 10.3390/antibiotics12020203 (PMC9952626; doi:10.3390/antibiotics12020203)
Supplement: Supplementary file 1 [file antibiotics-12-00203-s001.zip › antibiotics-2116643-supplementary.pdf]

Supplementary Table S1. Mass spectra of compounds identified in acetone extract of *Cullen tomentosum* using Liquid-Chromatography-Mass Spectrometry (LC-MS) and mz cloud library.

Structure of compounds	Name of compound (m/z cloud library)	Mass spectra
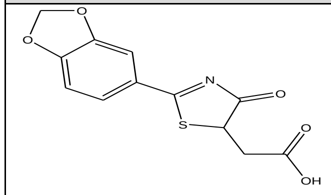	2-(2-(benzo[d][1,3] dioxol-6-yl)-4,5-dihydro-4-oxothiazol-5-yl)acetic acid	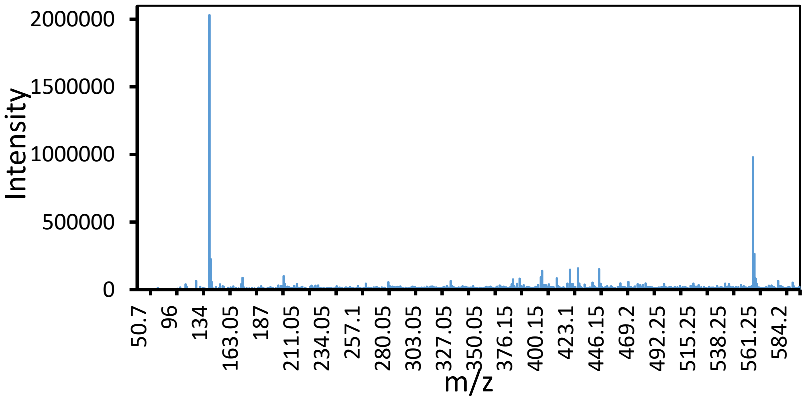
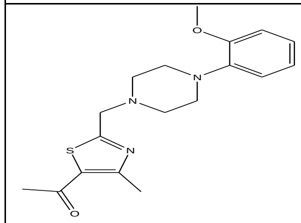	1-(2-((4-(2-methoxyphenyl) piperazin-1-yl) methyl) -4-methylthiazol-5-yl) ethanone	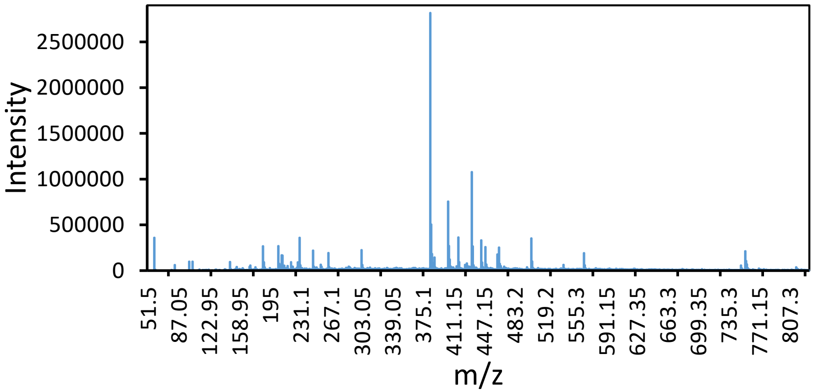

<table>
<tr><th>Structure of compounds</th><th>Name of compound (m/z cloud library)</th><th>Mass spectra</th></tr>
<tr><td>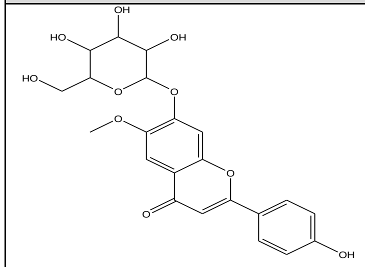</td><td>2-(4-hydroxyphenyl)-6-methoxy-7-(tetrahydro-3,4,5-trihydroxy-6-(hydroxymethyl)-2H-pyran-2-yloxy)-4H-chromen-4-one</td><td>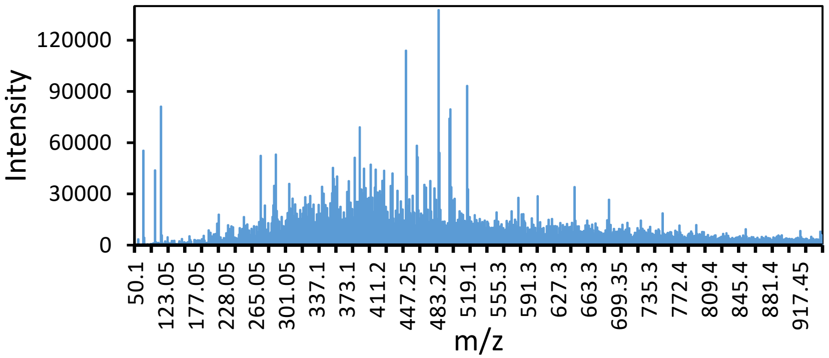</td></tr>
<tr><td>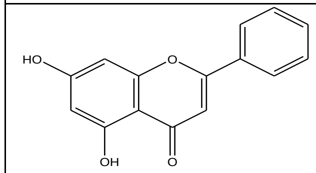</td><td>5,7-dihydroxy-2-phenyl-4H-chromen-4-one</td><td>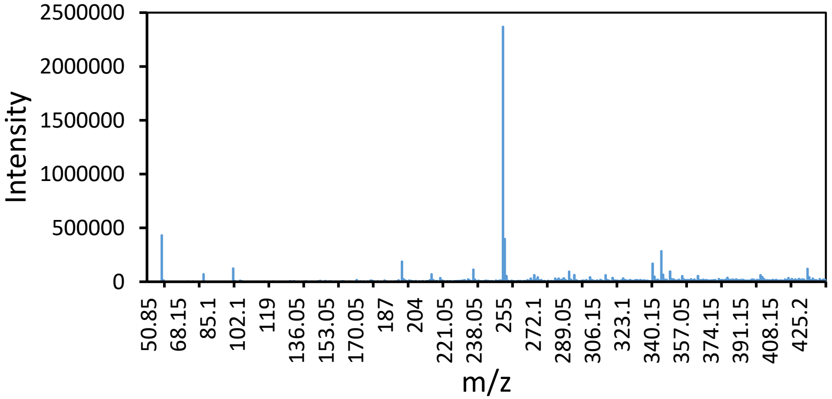</td></tr>
</table>

Structure of compounds	Name of compound (m/z cloud library)	Mass spectra
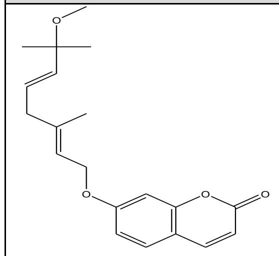	7-((2E,5E)-7-methoxy-3,7-dimethylocta-2,5-dienyloxy)-2H-chromen-2-one	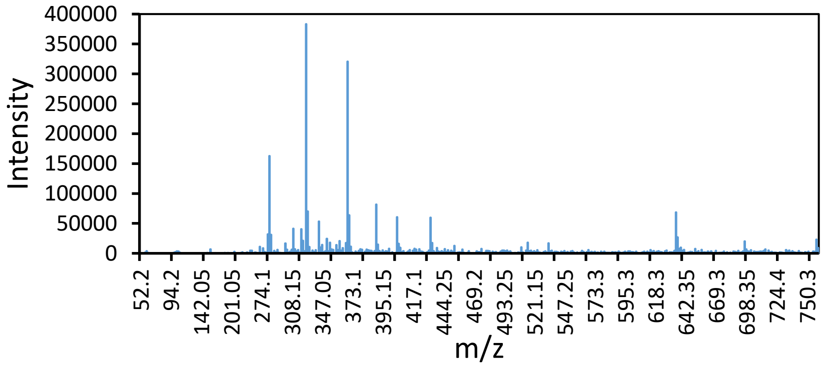
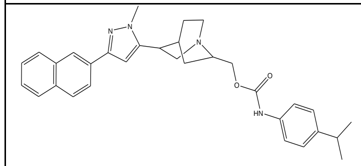	(3-(1-methyl-3-(naphthalen-6-yl)-1H-pyrazol-5-yl) quinuclidin-7-yl) methyl 4-isopropylphenylcarbamate	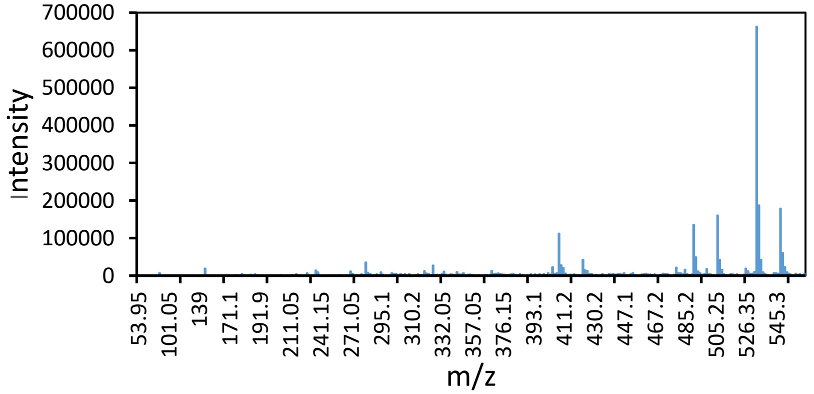

Structure of compounds	Name of compound (m/z cloud library)	Mass spectra
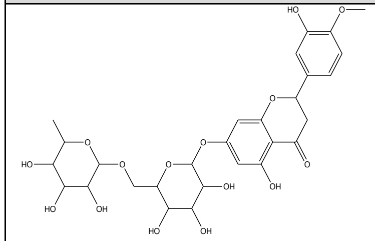	No name identity from NIST Library	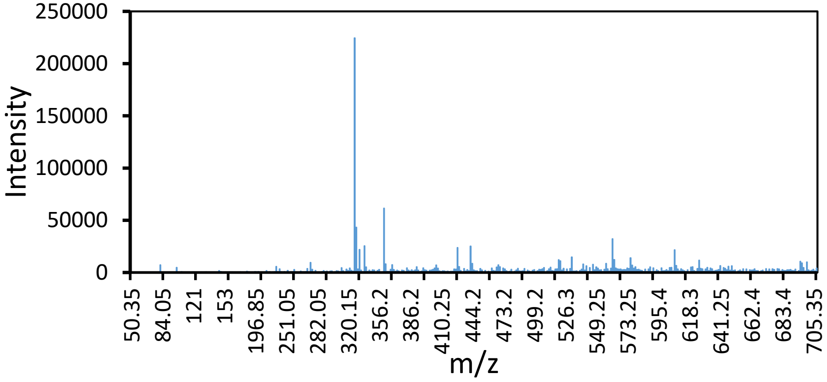
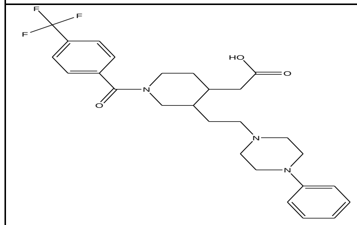	No name identity from NIST library	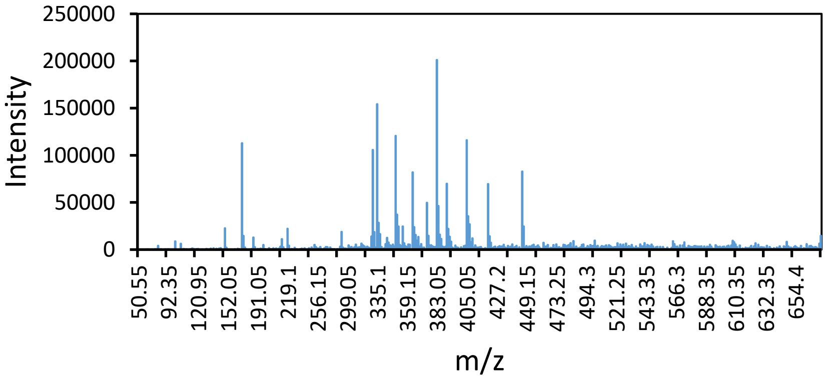

Structure of compounds	Name of compound (m/z cloud library)	Mass spectra
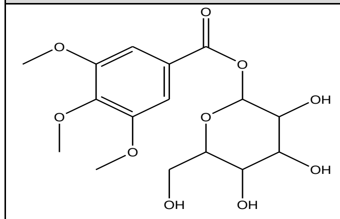	tetrahydro-3,4,5-trihydroxy-6-(hydroxymethyl)-2H-pyran-2-yl 3,4,5-trimethoxybenzoate	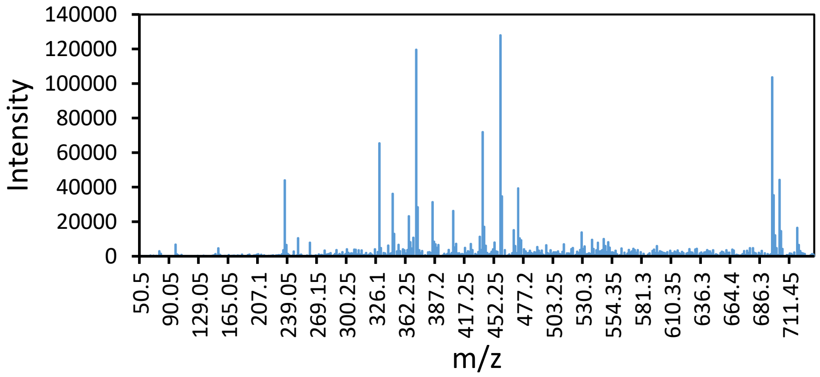
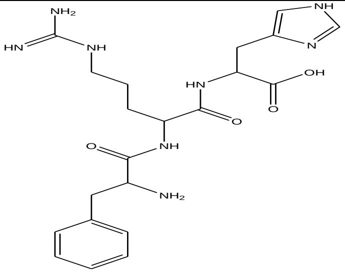	2-(guanidine)-3-(1H-imidazol-4-yl) propanoic acid	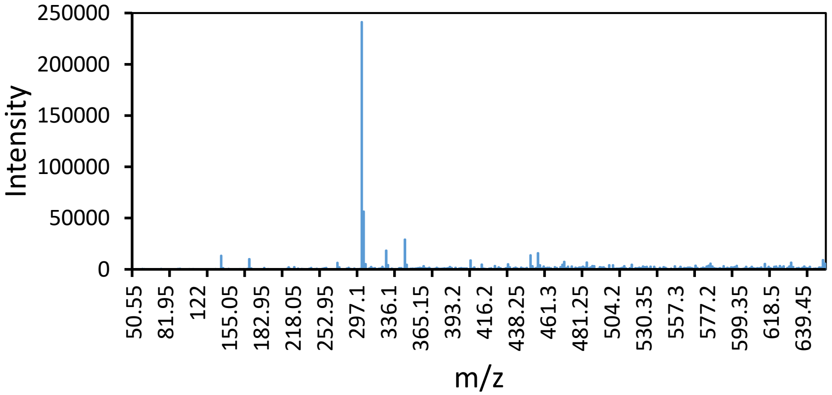

Structure of compounds	Name of compound (m/z cloud library)	Mass spectra
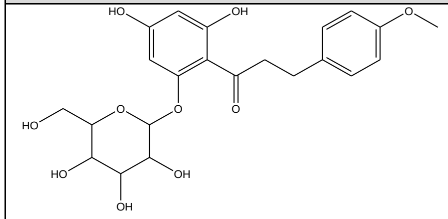	No name identity equivalent in NIST library	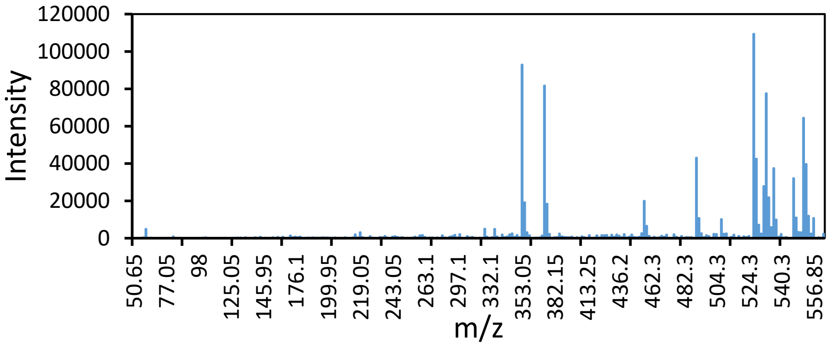
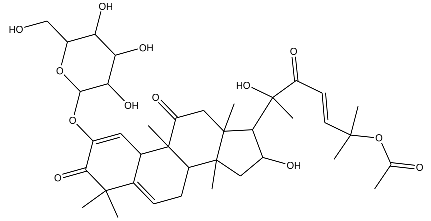	No name identity equivalent in NIST library	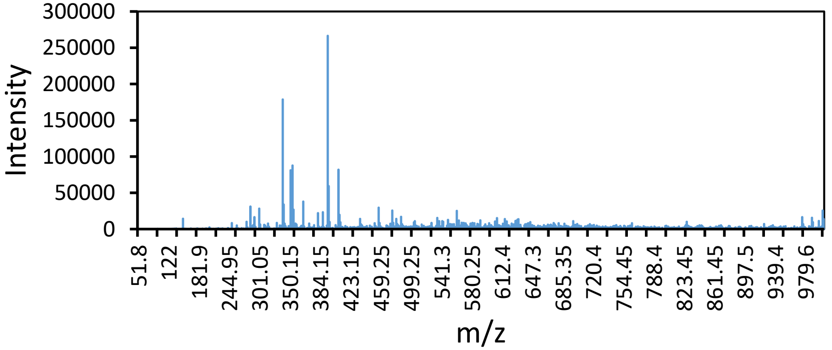

Structure of compounds	Name of compound (m/z cloud library)	Mass spectra
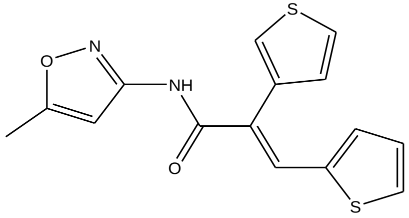	(E)-N-(5-methylisoxazol-3-yl)-3-(thiophen-2-yl)-2-(thiophen-3-yl) acrylamide	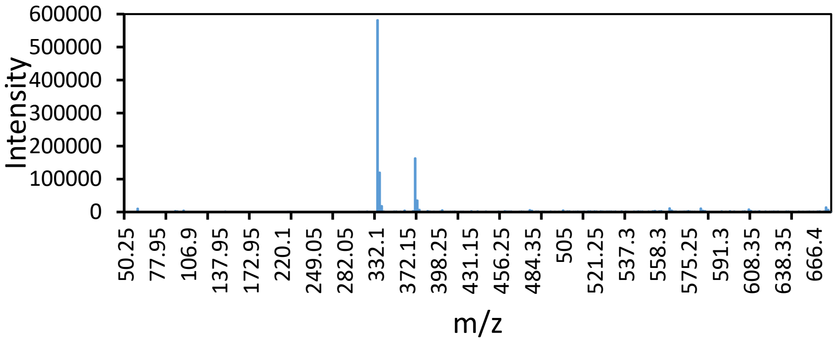
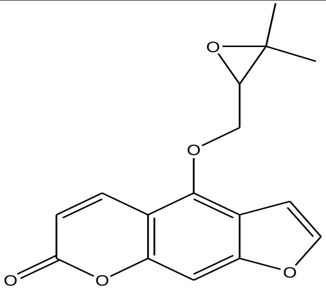	4-((3,3-dimethyloxiran-2-yl) methoxy)-7H-furo[3,2-g] chromen-7-one	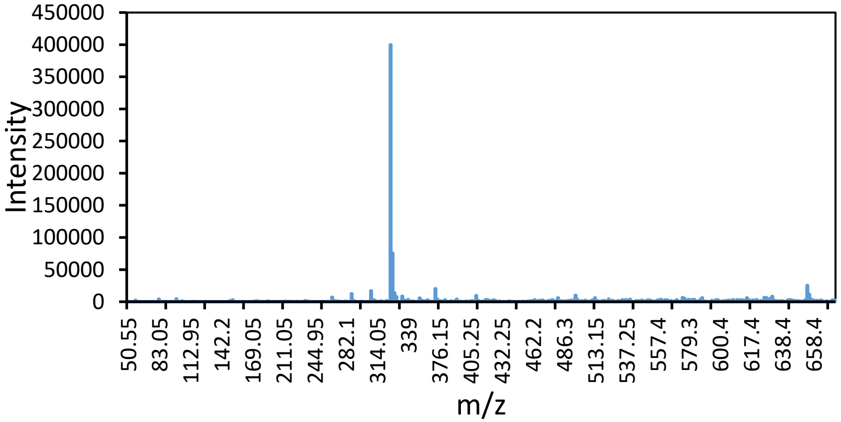

Structure of compounds	Name of compound (m/z cloud library)	Mass spectra
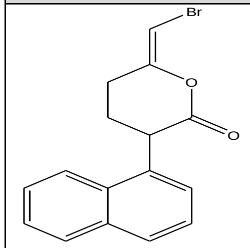	(Z)-6-(bromomethylene)-tetrahydro-3-(naphthalen-5-yl) pyran-2-one	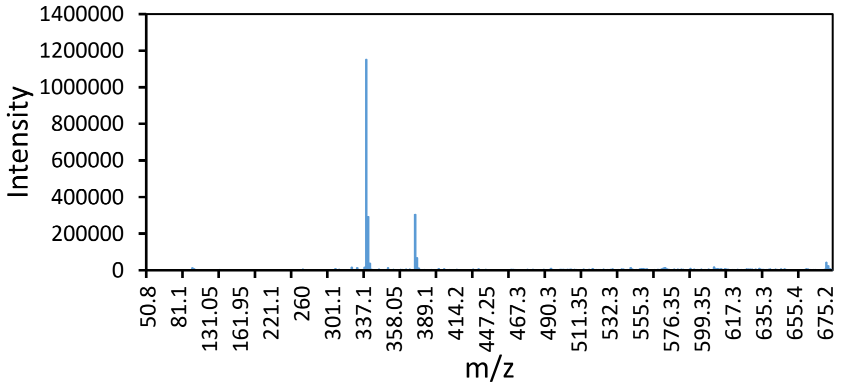
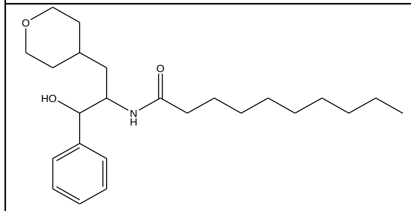	N-(3-(tetrahydro-2H-pyran-4-yl)-1-hydroxy-1-phenylpropan-2-yl) decanamide	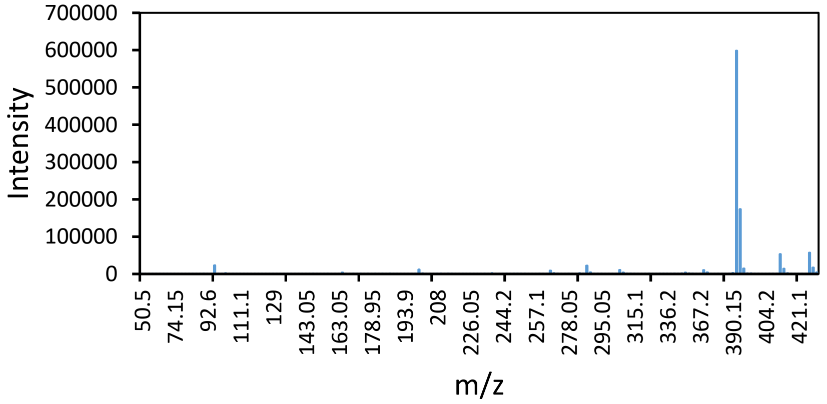

Structure of compounds	Name of compound (m/z cloud library)	Mass spectra
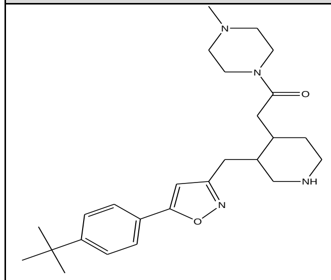	2-(3-((5-(4-tert-butylphenyl) isoxazol-3-yl) methyl) piperidin-4-yl)-1-(4-methylpiperazin-1-yl) ethanone	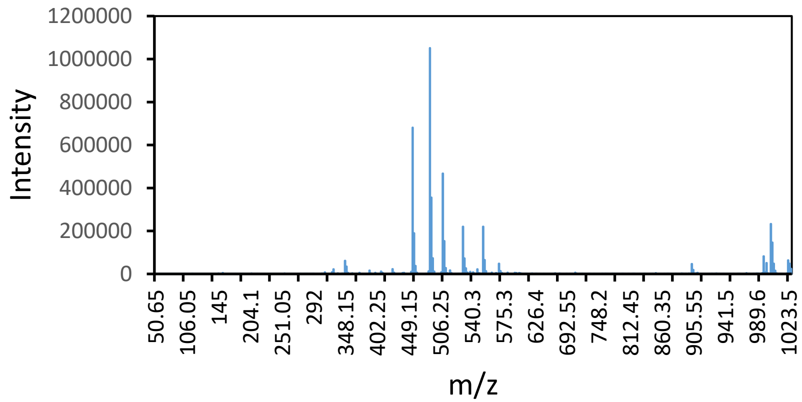
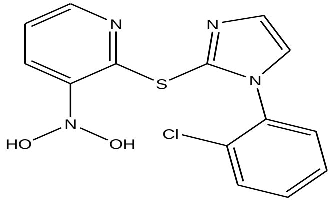	2-(1-(2-chlorophenyl)-1H-imidazol-2-ylthio)-N, N-dihydroxypyridin-3-amine	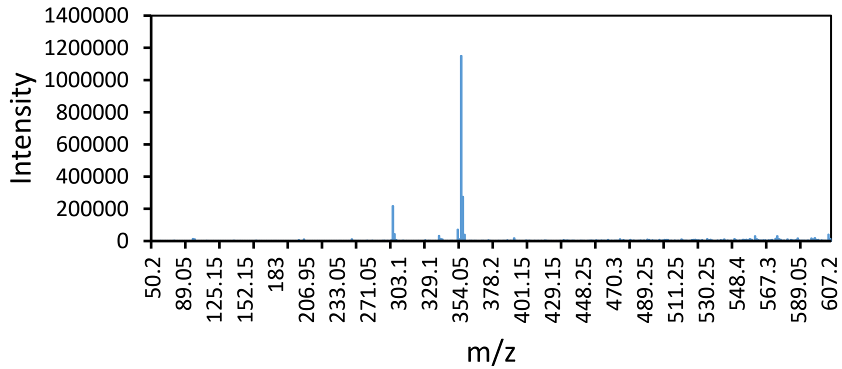

Structure of compounds	Name of compound (m/z cloud library)	Mass spectra
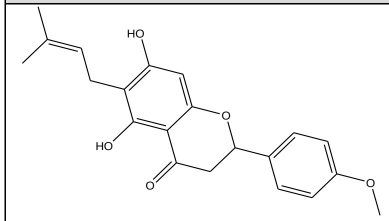	2,3-dihydro-5,7-dihydroxy-2-(4-methoxyphenyl)-6-(3-methylbut-2-enyl) chromen-4-one	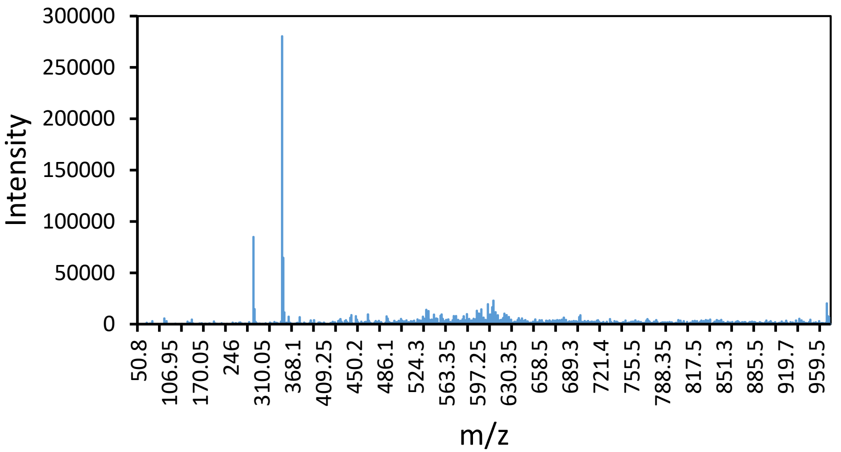

*RT = retention time, #PA = peak area.
